# Efficacy and Tolerability of Ziprasidone Use in Children and Adolescents, a Systemic Review and Meta Analysis

**DOI:** 10.1192/j.eurpsy.2022.1076

**Published:** 2022-09-01

**Authors:** A. Wadhwa, A. Sareen, C. Soeung, I. Penuelas-Calvo

**Affiliations:** 1 University of Alabama, Psychiatry, Birmingham, United States of America; 2 Bronx Care Health System-Affiliated with the Icahn School of Medicine at Mount Sinai, Psychiatry, New York, United States of America; 3 Bronx Care health System, Psychiatry, New York, United States of America; 4 Fundación Jiménez Díaz, Psychiatry, Madrid, Spain

**Keywords:** Ziprasidone

## Abstract

**Introduction:**

Ziprasidone is an atypical antipsychotic that has demonstrated efficacy for the treatment of bipolar disorder and schizophrenia. There is some preliminary evidence for Ziprasidone use in children and adolescents with several open label studies and some randomized control trials, therefore it is advantageous to understand where Ziprasidone lies in the treatment algorithm of children and adolescents.

**Objectives:**

The aim of our study is to examine the efficacy and tolerability of Ziprasidone in children and adolescents.

**Methods:**

We conducted a literature search consisting of open label or randomized control trials (RCT) that report on Ziprasidone use in children on the PubMed database. We found 13 studies (11 open label and 2 RCT) that met our inclusion criteria. Our outcome measures included efficacy measures such as BPRS, YMRS, CGI-S and adverse effects such as weight gain, increase in BMI, QTc prolongation, sedation, dizziness and EPS.

**Results:**

Data from thirteen studies was meta-analyzed (Total n= 560, mean age=13.16 years, male= 70.35% that reported the use of Ziprasidone in children and adolescents. We found that Ziprasidone was efficacious in children and adolescents in measures of BPRS (-13.493, p<0.05), YMRS (-14.225, p<0.05), CGI-S (-1.430, p<0.05). In measures of adverse effects, Ziprasidone was not found to cause any significant weight gain (0.164, p>0.05) or change in BMI (-0.159, p>0.05). QTc prolongation was found to be significant (13.122, p<0.05).

**Conclusions:**

Ziprasidone is an efficacious in children and adolescent population. It does not cause significant weight gain, however QTc prolongation and sedation were found to be the most significant side effects .

**Disclosure:**

No significant relationships.

